# Effects of ionizing radiation and different resin composites on shear strength of ceramic brackets: an in vitro study

**DOI:** 10.1590/2177-6709.27.2.e2219330.oar

**Published:** 2022-06-10

**Authors:** Amadeu TOMASIN, Flávia AMARAL, Fábio ROMANO

**Affiliations:** 1 Universidade Estadual do Oeste do Paraná, Departamento de Odontologia, Disciplina de Ortodontia (Cascavel/PR, Brazil).; 2 Faculdade São Leopoldo Mandic, Centro de Pesquisas São Leopoldo Mandic, Departamento de Odontologia Restauradora (Campinas/SP/Brazil).; 3 Universidade de São Paulo, Faculdade de Odontologia de Ribeirão Preto, Disciplina de Ortodontia (Ribeirão Preto/SP, Brazil).

**Keywords:** Dental enamel, Ionizing radiation, Shear strength, Orthodontics

## Abstract

**Introduction:**

Head and neck cancer is considered one of the most common types, and its treatment includes radiotherapy, which can trigger side effects and undesirable sequelae in the oral cavity and dental tissues.

**Objective:**

This study aimed to make an *in vitro* evaluation of the shear strength and failure mode of ceramic orthodontic brackets bonded with two different composites in enamel submitted to ionizing radiation.

**Methods:**

After the study was approved by the Research Ethics Committee, 60 healthy human premolars were selected and divided into two groups, based on the absence or presence of ionizing irradiation of the enamel. The fragments were thermocycled and then randomly subdivided into two subgroups, according to the composite used for bonding the ceramic brackets (Inspire Ice - Ormco) to the enamel (n = 15): Transbond XT composite (3M), and Light Bond composite (Reliance). After 24 hours, the specimens were submitted to the shear strength test, and the failure mode was analyzed using a stereomicroscope and confocal microscopy. The shear strength data were submitted to two-way ANOVA, considering a significance level of 5%.

**Results:**

The groups submitted to radiation presented lower shear strength values (4.48MPa) than those not irradiated (9.23MPa) (*p*< 0.001), and the tested composites were not statistically different (*p*= 0.078). Regarding the fracture mode, all the groups presented mostly adhesive fractures.

**Conclusion:**

It was concluded that ionizing radiation negatively affects the adhesion of ceramic brackets, regardless of the composite used for bonding.

## INTRODUCTION

Cancer treatment is administered with ionizing radiation, which destroys neoplastic tissues, interacts with tissues, and forms electrons that ionize the environment, create chemical effects by water hydrolysis, and disrupt DNA strands.^1^ However, treatment with radiotherapy may have undesirable side effects and sequelae in the oral cavity.^2^ ^,^ ^3^ In addition to these side effects, changes may occur in the physical and adhesive properties of dental enamel following ionizing radiation.^4^ ^,^ ^5^ These changes should be considered by the dental surgeon when performing restorative procedures, and bonding orthodontic brackets.^5^

The advancement of cancer treatment methods, and the early diagnosis of the disease^6^ ^-^ ^10^ have substantially improved the survival and cure rates in recent decades. Therefore, more and more dentists are subject to treat patients who have undergone cancer treatment, such as radiotherapy. This increase in cancer treatments demands greater attention on the changes that may be occurring in the oral cavity, both in soft and hard tissues.^11^ In cases where radiotherapy is applied to the head and neck region, enamel and dentin may present structural changes, and modifications in their physicochemical properties.^4^ ^,^ ^12^ ^-^ ^17^

Despite the increasing use of ceramic brackets for orthodontic treatments, few studies in the literature have evaluated how ionizing radiation interferes with the bonding of these brackets to dental enamel^5^ ^,^ ^18^. Thus, this study aimed to make an *in vitro* evaluation of the shear strength and failure mode of ceramic orthodontic brackets bonded with two different enamel composites submitted to ionizing radiation. The null hypothesis tested is that there would be no difference in the shear strength of different composites used for bonding ceramic brackets, in either irradiated or non-irradiated enamel.

## MATERIAL AND METHODS

After the study was approval by the Research Ethics Committee of *Faculdade de Odontologia de Ribeirão Preto, Universidade de São Paulo* (CAAE #5425.4816.5.0000.5419), 60 teeth were selected from maxillary and mandibular premolars extracted according to the orthodontic planning of patients of both sexes, with mean age of 14 years and 5 months. The teeth showed no wear facets, and had complete rhizogenesis, thereby enabling standardization by age and maturation of dental enamel.

### EXPERIMENTAL DESIGN

This study evaluated *in vitro* the following two factors:

1) Dental enamel radiation, as: present or absent.
2) Composite used for bracket bonding, being:
  a) Transbond XT (3M Unitek, Monrovia, CA, USA);
  b) Light Bond (Reliance Orthodontic Products, Itasca, IL, USA).

The association between the factors under study resulted in four experimental, randomly divided groups (Table 1). Each experimental unit was composed of a bracket/tooth set (n = 15). The sample size was calculated considering the shear strength variable, and based on the Analysis of Variance (ANOVA) test, with a significance level of 5% (α = 0.05), and a test power of 80%, resulting in 60 specimens (n = 15) (G Power 3.1 software, University of Düsseldorf, Germany). The response variables were shear strength results in MPa, failure mode analysis, and adhesive interface evaluation.

**Table 1:** Groups analyzed in this study.

Irradiation	Composite	Group identification	n	Total
Present (I)	Transbond XT	GTI	15	60
Absent (NI)	Transbond XT	GTNI	15	
Present (I)	Light Bond	GLI	15	
Absent (NI)	Light Bond	GLNI	15	

GTI = Group Transbond irradiated; GTNI = Group Transbond non-irradiated; GLI = Group Light Bond irradiated; GLNI = Group Light Bond non-irradiated.

### MAKING OF THE SPECIMENS

The procedures for tooth extraction and sample preparation of the dental adhesive systems followed the recommended guidelines set by the International Organization for Standardization (ISO, TR 11450). The specimens were made to undergo the shear test and fracture mode analysis after bracket detachment. The procedure consisted of covering the dental crown of the premolars with pink self-curing acrylic resin, and then pressing the buccal face of each tooth into a glass plate before the polymerization reaction was triggered. Next, the faces were prepared with #400, #600 and #1200 grit sandpaper using a polishing machine (DP-902 polisher, Struers A/S, Copenhagen, Denmark), until the enamel area for bracket bonding was planed to approximately 5 mm^2^, to enable bracket adhesion.

A device with 4-mm glass plates was made to contain the premolars in PVC tubes. It consisted of:

1. a base plate;
2. two perpendicular plates fixed to the sides;
3. a horizontally fixed plate covering half of the base plate;
4. a perpendicular plate fixed to the side plates.

Subsequently, the premolar roots were introduced into and centered in PVC tubes approximately 1.5-cm high, filled with self-curing acrylic resin, and the excess resin was removed with a Lecron spatula (Duflex, Juiz de Fora/MG, Brazil). This device not only enabled the tooth roots to be introduced into the PVC tubes, but also helped maintain the tooth perpendicular to the base of the tube. This positioning is essential to provide parallelism between the exposed enamel face and the shear chisel during the mechanical test, given that any change in this angle may alter the test result.

### IRRADIATION PROCEDURE

Half of the sample (30 teeth) was irradiated at the Radiation Therapy Center at the *Universidade de São Paulo, Faculdade de Medicina* (HCFMRP-USP, Ribeirão Preto/SP, Brazil). The other half was stored in distilled water at 4^o^C. During the irradiation procedure, the experimental specimens were placed in a plastic box immersed in deionized water, to keep them in a humid environment, simulating the oral cavity. At the end of the procedure, the deionized water was discarded, and the specimens were kept in artificial saliva in an incubator at 37ºC, until the next irradiation procedure, at which time the saliva was again replaced by deionized water. The artificial saliva was discarded before irradiation because of its high concentration of ions, which could interfere with the direct radiation per unit area. 

The specimens were subjected to a fractioned dose of 2 Gy over five consecutive days, until reaching the total 60 Gy dose for all 30 fractions after six weeks (Table 2)^4^ ^,^ ^15^ ^,^ ^17^ ^,^ ^19^ ^,^ ^20^. The X-rays were emitted from an irradiator designed for biological research (RS 2000, Rad Fonte Technologies, Suwanee, GA, USA), with a power of 200 kVp and 25 mA, and a standard 0.3-mm copper filter. X-rays generated under these conditions have a spectrum of minimum and maximum energy values ranging from 95 kV to 200 kV, and a half value layer of 0.62 mm of copper. The dose gradient of these X-rays in tissue is about 10% at 0.5-mm deep. The plates were aligned equidistant from the beam center and inner cone, to ensure a uniform dose rate (approximately 2.85 Gy/min), and total dose delivery per fraction. Quality control was performed using Nanodot dosimeter (Landauer, Glenwood, IL, USA) with plate surface dose readings used to calculate beam-on treatment time. The dosimeters were placed below the irradiated plates and calibrated according to the beam conditions described above. After the ionizing radiation was terminated, the specimens were kept in artificial saliva in an incubator at 37^o^C for 24 hours.

**Table 2:** Number of irradiation cycles, periods and total doses delivered.

Number of irradiation cycles	Period (in days)	Total dose (2 Gy/cycle)
5	5	10 Gy
10	10	20 Gy
15	15	30 Gy
20	20	40 Gy
25	25	50 Gy
30	30	60 Gy

### ACCELERATED ARTIFICIAL AGING

All the teeth underwent accelerated artificial aging, and were again placed in artificial saliva for one week. This aging was performed by thermocycling (Biocycle, Biopdi SA, São Carlos/SP, Brazil) to simulate the one-year period suggested in the literature as the minimum time required to start orthodontic treatment of a patient after radiotherapy. This cycle is assumed to occur 20 to 50 times a day *in vivo*. Thus, the specimens were submitted to 10,000 cycles, with baths at a temperature ranging from 5°C to 55ºC, simulating approximately the one-year period^21^. 

### ORTHODONTIC BRACKET BONDING

Before the ceramic orthodontic brackets (Inspire ICE, Ormco, Orange, CA, USA) were bonded in the experimental and control groups, an area of 5mm^2^ in diameter was delimited on the buccal surface of the premolar crowns, using adhesive tape with a central hole. The Inspire ICE brackets used in this study are made from monocrystalline ceramic and have tiny spherical particles at their base, providing increased retention and reduced fracture propagation.

This area then received prophylaxis with extra-fine pumice paste and water, and a rubber prophy cup at low-speed for 10 seconds, after which it was washed for 10 seconds, and dried with a triple syringe free of oil and moisture for the same period. Each rubber cup was used on only five teeth, thus preventing rubber wear from impairing the prophylaxis efficiency. Immediately afterwards, the enamel was conditioned with 37% phosphoric acid for 15 seconds, followed by vigorous washing with air/water jet for 10 seconds, and drying for the same period.

Enamel fragments with or without irradiation were divided into two subgroups, according to the bonding composite: Transbond XT composite (3M Unitek, Monrovia, CA, USA), and Light Bond composite (Reliance Orthodontic Products, Itasca, IL, USA). Table 3 describes the composition of the materials.

**Table 3:** Composite resins used in the experiment and their respective composition.

Composites (manufacturer)	Composition
Transbond XT (3M Unitek, Monrovia, CA, USA)	BIS-GMA, TEGDMA, silane treated silica, n-dimethyl benzocaine, hexafluorophosphate, camphorquinone
Transbond XT Primer (3M Unitek, Monrovia, CA, USA)	BIS-GMA, 4-Dimethylamino, benzene ethanol, camphorquinone, hydroquinone
Light Bond (Reliance Orthodontic Products, Itasca, IL, USA)	UDMA, BIS-GMA, fused silica, and a component not declared by the manufacturer
Light Bond Sealant (Reliance Orthodontic Products, Itasca, IL, USA)	UDMA, BIS-GMA, TEGDMA, tetrahydrofurfuryl methacrylate, fluoride

BIS-GMA - Bisphenol A Diglycidyl methacrylate; TEGDMA - Triethylene Glycol Dimethacrylate; UDMA: Urethane Dimethacrylate.

In relation to the groups that received Transbond XT composite, a thin layer of XT Primer bonding agent (3M Unitek, Monrovia, CA, USA) was applied prior to bonding the brackets, spread evenly over the enclosed area with a light air jet, and photoactivated for 10 seconds with a Ultra Blue LED light (DMC, Plantation, FL, USA). Transbond XT composite (3M Unitek, Monrovia, CA, USA) (Table 3) was then applied to the base of the brackets, which were positioned and pressed with a grasping forceps (Ortoply, Philadelphia, PA, USA) into the delimited vestibular areas. The bonding procedure was performed by a single, calibrated operator, in order to standardize the pressure applied to the brackets. Excess composite around the brackets was removed with a blunt-ended probe explorer, and then photoactivated on the mesial, distal, incisal, and cervical faces for 5 seconds on each face. The light intensity of the device was measured with a radiometer (Demetron, Danbury, CT, USA) at every four photoactivations, maintaining a light intensity of 600 mW/cm^2^.

In groups that received Light Bond composite, a thin layer of Light Bond Sealant Resin (PRO SEAL, Reliance Orthodontic Products Itasca, IL, USA) was applied to the enamel of the previously delimited buccal face. Then, a light air jet was applied to spread the bonding agent evenly, after which photoactivation was performed for 10 seconds. The brackets were bonded with Light Bond composite (Reliance Orthodontic Products Itasca, IL, USA) and photoactivated in the same way as in Transbond XT groups.

After the brackets were bonded, the specimens were kept intact for 30 minutes, and then stored in artificial saliva for 24 hours in an incubator at 37^o^C, until the mechanical test was performed. Alterations resulting from curvature of the base were avoided by using only brackets indicated for mandibular incisors, because they have a flat base and are better to position the chisel properly during the mechanical shear test.

### SHEAR STRENGTH TEST

The specimens were then placed in a mechanical testing machine (Instron, Model 2519-106, Canton, MA, USA), and the brackets were detached at a speed of 0.5 mm/min with a 20 Kgf load cell. The active chisel tip was supported at the composite/enamel interface, and the force was applied until the moment of rupture, at which time the maximum force value of the movement was recorded. The shear bond strength was calculated by dividing the maximum force recorded during the test (in N) by the area of the ceramic brackets (obtained by the manufacturer) - the values were expressed in MPa.

### FAILURE PATTERN ANALYSIS

A confocal laser microscope (LEXT Olimpus, Japan) and OLS 400^R^ software were used to perform the fracture type analysis. Images were evaluated with a 5X objective, at a final magnification of 107x. Nine samples from GTNI, 10 from GTI, 8 from GLNI, and 10 from GLI were evaluated.

The images were categorized into the following fracture modes according to the literature^22^ ^,^ ^23^:

*Adhesive fracture (Ad)* - the fracture occurs between the composite and the enamel.
*Resin cohesive fracture (CR)* - the fracture occurs between the bracket and the composite, leaving the composite adhered to the enamel.
*Enamel cohesive fracture (CE)* - the fracture occurs on the dental surface, causing removal of part of the enamel.
*Resin/adhesive cohesive mixed fracture (CR/Ad)* - the fracture occurs between the bracket and the composite on the same specimen; part of the composite remains on the dental surface, and the other part is fractured at the enamel interface.
*Enamel/adhesive cohesive mixed fracture (CE/Ad)* - the dental structure is partially removed on the same specimen, and the remaining structure is fractured between the composite and the enamel.
*Resin cohesive/Enamel cohesive mixed fracture (CR/CE)* - part of the composite remains on the dental surface of the same specimen, and the dental structure is partially removed.

**Figure 1:**
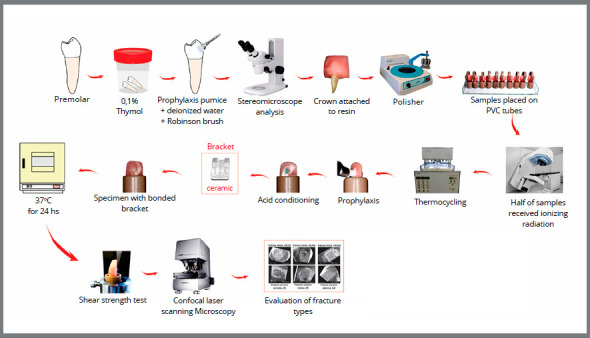
Methodology used in the present study.

The flowchart presented in the Figure 1 shows the main methodological procedures of this study.

### DATA ANALYSIS

Compliance with normality and homoscedasticity assumptions were checked using the Shapiro-Wilk and Levene tests, respectively, and the effects of the ionizing radiation, the composite type, and the interaction of these two study factors were investigated. The shear bond strength data of the ceramic brackets to enamel was submitted to two-way analysis of variance. Error of method for the failure pattern analysis was performed by randomly selecting 20% of the specimens and measuring them again four weeks after the first measurement (Wilcoxon Sign Ranked test = 0.876). Statistical calculations were performed using the SPSS 23 program (SPSS, Chicago, IL, USA), adopting a significance level of 5%.

## RESULTS

### SHEAR STRENGTH TEST

**Figure 2:**
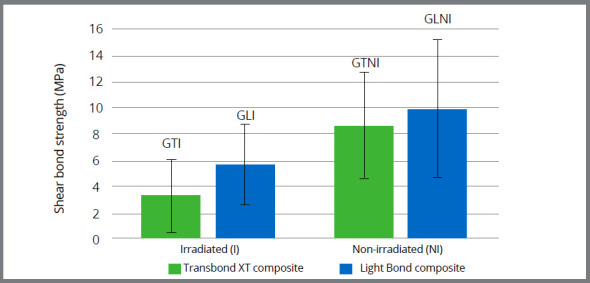
Column diagram of the average bond strength values of ceramic brackets bonded with different composite resins on enamel submitted or not to ionizing radiation. GTI = Group Transbond irradiated; GLI = Group Light Bond irradiated; GTNI = Group Transbond non-irradiated; GLNI = Group Light Bond non-irradiated.

The two-way analysis of variance showed that the ionizing radiation significantly affected the shear bond strength of ceramic brackets to enamel (*p* < 0.001). Table 4 shows that the ionizing radiation reduced the bond strength values of brackets bonded with Transbond and Light Bond composite resins by 42.7% and 61.16%, respectively. The comparing the bond strength values using the two composites showed that there were no statistical differences between Transbond XT and Lightbond composites (*p* = 0.078, Table 4 and Fig 2).

**Table 4:** Mean values and standard deviation of the bond strength (MPa) of ceramic brackets bonded with different resins composed of enamel submitted or not submitted to ionizing radiation.

Ionizing radiation	Composite		Overall average
	Transbond	Light Bond	
Present	3.30 (2.74)	5.65 (3.04)	4.48 (3.09)^B^
Absent (control)	8.59 (4.09)	9.87 (5.26)	9.23 (4.68)^A^
Overall average	5.94 (4.35)*	7.76 (4.73)*	--

Overall averages followed by different uppercase letters (A/B) indicate a statistically difference between irradiated and no irradiated enamel, regardless of composite.

Overall averages followed by asterisks indicate absence of statistical difference between composites, regardless of the presence of irradiation or not (ANOVA, *p* < 0.05).

### FRACTURE PATTERN ANALYSIS

**Figure 3:**
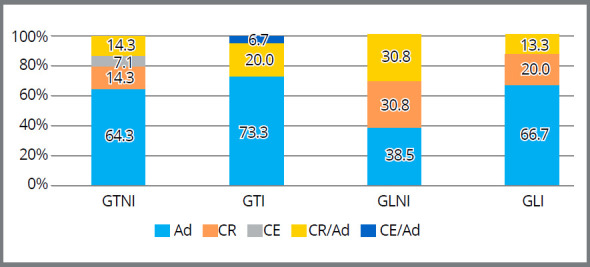
Mean fracture patterns, percentage, after shear test. Ad = Adhesive fracture; CR= Resin cohesive fracture; CE = Enamel cohesive fracture; CR/Ad = Resin/adhesive cohesive mixed fracture; CE/Ad = Enamel/adhesive cohesive mixed fracture. GTI = Group Transbond irradiated; GLI = Group Light Bond irradiated; GTNI = Group Transbond non-irradiated; GLNI = Group Light Bond non-irradiated.

Analysis of fracture patterns showed that the adhesive fracture was predominant in all groups. However, there was a high percentage of cohesive fracture in resin (CR) and resin/adhesive cohesive mixed fracture (CR/Ad) in the non-irradiated group, in which brackets were bonded with Light Bond composite (Fig 3). 

### MORPHOLOGICAL ANALYSIS

**Figure 4:**
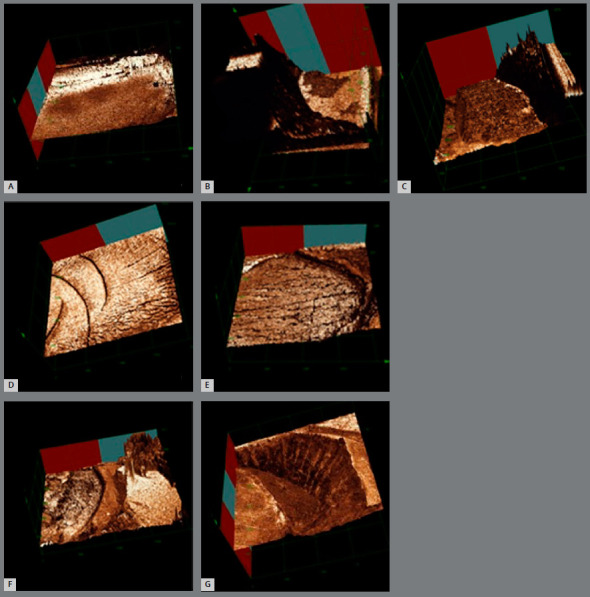
A) Transbond non-irradiated (adhesive fracture). B) Light Bond non-irradiated (cohesive in resin fracture). C) Transbond irradiated (cohesive in enamel fracture). D) Light Bond irradiated (Mixed fracture - cohesive in resin and adhesive). E) Transbond irradiated (mixed fracture - cohesive in enamel and adhesive). F) Light Bond irradiated (mixed fracture - cohesive in resin and cohesive in enamel). G) Transbond irradiated (cohesive fracture in enamel).

Representative images of each failure mode can be seen in Figure 4. 

## DISCUSSION

Structural changes in enamel and dentin from ionizing radiation present as decreased or increased microhardness, increased solubility, reduced stability of the dentin-enamel junction, altered enamel prism structure, increased calcium concentration, and reduced oxygen in the enamel.^12^ ^-^ ^15^ ^,^ ^24^ Thus, dental surfaces altered by radiotherapy become susceptible to adhesive failures, such as failures related to bracket bonding used in corrective orthodontic treatment.^4^ ^,^ ^17^ ^,^ ^20^ ^,^ ^25^ ^,^ ^26^

Adequate adhesion of brackets is essential, whether metallic or ceramic, since frequent rebonding prolongs the time of corrective orthodontic treatment and hinders orthodontic mechanics.^25^ The results of the present study investigating the adhesion of brackets on irradiated teeth, based on the shear strength test, show that the ceramic brackets bonded to the teeth submitted to ionizing radiation presented significantly lower bond strength values. Similarly, Santin et al.^5^ concluded that the adhesion of metallic and ceramic orthodontic brackets bonded to tooth enamel submitted to irradiation presented lower shear strength values.

These results are corroborated by the studies on the adhesion of composites in irradiated teeth^26^ ^-^ ^28^ that showed a decrease in the adhesive strength to similar dental surfaces after radiotherapy. Thus, it is suggested that ionizing radiation negatively affects enamel bond strength when the procedure is performed after radiotherapy. The result of the bond strength is in line with the failure mode, as the adhesive fracture was predominant in all the study groups evaluated, and was higher in the group in which enamel was irradiated. 

The shear strength values presented in this study for brackets bonded with Light Bond composite on the non-irradiated dental surface (9.87 MPa) are similar to those presented in the literature for this same composite.^29^ ^-^ ^31^ Likewise, the shear strength values found for Transbond XT composite (8.59 MPa) in the present study are similar to those found in the literature for this same material.^5^ ^,^ ^29^ ^-^ ^31^ The bond strength values of the composites in non-irradiated enamel, evaluated in the present study, are within the standard of those considered clinically useful. According to Reynolds and von Fraunhofer,^32^ these values are between 5.6 and 6.8 MPa, that is to say, values that would successfully resist orthodontic and chewing forces. Thus, based on the values found for the non-irradiated enamel, it can be inferred that only the group in which the brackets were bonded to the irradiated enamel with Light Bond composite presented values compatible with clinical use (5.65 MPa), whereas brackets bonded with Transbond XT composite were below the reference values (mean value of 3.3 MPa). Therefore, it could be further suggested that Light Bond composite promotes better bond strength to irradiated enamel, within acceptable clinical parameters. This result explains the failure mode found in Transbond groups, which showed not only adhesive failures, but a high prevalence of cohesive resin and mixed failures (resin cohesive/adhesive). Hence, the adhesive interface between the composite resin and the enamel was better preserved. 

Based on the findings of this study, it was observed that decreasing the adhesiveness of brackets bonded to irradiated enamel may hinder but not preclude orthodontic mechanics. Orthodontic mechanics with lighter force levels should be preferred for patients undergoing head and neck radiotherapy. A clinical study should be performed to evaluate the failure rates.

## CONCLUSION

The bond strength of ceramic composites and brackets to enamel subjected to ionizing radiation was reduced.

There were no significant differences between Transbond XT and Light Bond composites, regardless of the presence of ionizing irradiation to enamel or not.

There was a predominance of adhesive fractures for all the groups evaluated, indicating that the fracture occurred between the enamel and the composite.
